# An Internet of Things (IoT) Application on Volcano Monitoring

**DOI:** 10.3390/s19214651

**Published:** 2019-10-26

**Authors:** Shadia Awadallah, David Moure, Pedro Torres-González

**Affiliations:** Centro Geofísico de Canarias, Instituto Geográfico Nacional, C/La Marina 20, 38001 S/C Tenerife, Spain; admoure@fomento.es (D.M.); patorres@fomento.es (P.T.-G.)

**Keywords:** IoT, LoRa, Raspberry Pi, wireless sensor networks, thermometers, volcano monitoring

## Abstract

In the last few years, there has been a huge interest in the Internet of Things (hereinafter IoT) field. Among the large number of IoT technologies, the low-power wide-area network (hereinafter LPWAN) has emerged providing low power, low data-rate communication over long distances, enabling battery-operated devices to operate for long time periods. This paper introduces an application of long-range (hereinafter LoRa) technology, one of the most popular LPWANs, to volcanic surveillance. The first low-power and low-cost wireless network based on LoRa to monitor the soil temperature in thermal anomaly zones in volcanic areas has been developed. A total of eight thermometers (end devices) have been deployed on a Teide volcano in Tenerife (Canary Islands). In addition, a repeater device was developed to extend the network range when the gateway did not have a line of sight connection with the thermometers. Combining LoRa communication capabilities with microchip microcontrollers (end devices and repeater) and a Raspberry Pi board (gateway), three main milestones have been achieved: (i) extreme low-power consumption, (ii) real-time and proper temperature acquisition, and (iii) a reliable network operation. The first results are shown. These results provide enough quality for a proper volcanic surveillance.

## 1. Introduction

Volcanic eruptions are natural phenomena that can significantly affect populations in their immediate surroundings and even at greater distances. These phenomena occur when an over-pressurized batch of magma is able to open a path through the host rock and reach the surface. This movement of over-pressurized magma usually causes physical and chemical changes that mainly consist of increases in seismicity, gas emissions, surface deformation, and changes in other potential fields (gravity, magnetism, etc.) [1,2,3,4]. Measuring these geophysical and geochemical signals can help to identify any deviation from the background volcanic activity, making it possible to forecast where and when an eruption could take place. Therefore, a proper volcanic monitoring program is absolutely necessary in order to improve the understanding of a volcanic system [5,6]. Since manifestations of volcanic activity are so diverse, suitable volcano surveillance must always combine different techniques such as seismology [7], geodesy [8], and geochemistry [9]. 

More than 90% of the magmatic gases emitted in a volcanic area are water steam, followed in abundance by CO_2_, H_2_S, and SO_2_ [10,11,12]. These hot gases flow through the system of fractures interacting with the aquifers and rocks, changing their chemical and physical properties. At the surface, fumaroles and thermal anomalies zones indicate the main ways out of these deep hot gases. Since the chemical composition of the gases that is present in a volcano emission is a reflection of the activity [13], variations in their emission rates, composition, and temperature may indicate a change in the volcanic system. 

Temperature monitoring of fumaroles and thermal anomalies can be made in situ and/or remotely. In situ measurements are carried out by direct contact sensors. The instrumentation employed depends on the range of the expected temperature values and the thermal area features. Usually, thermocouples and thermistors are the main direct contact sensors employed when measuring temperature at volcanic areas. These sensors have to be adequately protected against the corrosion of acid gases present at these sites. Although direct measurements are very precise, they are limited to cover a specific area or measuring individual points. 

Temperature monitoring can also be made remotely by measuring the thermal infrared radiation (TIR) emitted by a heated surface. Nevertheless, TIR can be partially or completely obscured by thick clouds, dust, water vapor, and/or ash plumes, so these types of measurements should preferably be made during favorable atmospheric conditions. A common instrument is the infrared camera, whether fixed or portable [14]. This device estimates the surface temperature by means of the infrared radiation measurement in each pixel over a specific wavelength range. Then, all measures are merged, forming an image using a false color palette in order to identify possible thermal anomalies. This is a useful tool in volcano monitoring, since it allows measuring temperature from a safe distance. However, due to the usually large areas that are recorded, the spatial resolution of images is low. 

The mentioned procedures imply either to make the measurement in situ or to use non-portable and costly systems. Considering this, the possibility for instrumental development that offers alternatives to the presented drawbacks remains open. Although in recent years different works framed in this context have been published [15,16], there is still a very broad field in which to investigate. It is important to note that volcanic areas are usually remote and inaccessible with extreme environments, offering difficulties for communications, power supply, and maintenance. Thus, a compromise between the quality and consistency of retrieved data and the quantity of data delivered by the network, together with the cost and energetic requirement of the instrumentation must be reached. It would be desirable to develop portable, inexpensive (offering easy replacement), and low power consumption (since batteries or solar panels are often used as power supplies) instrumentation for monitoring the geochemical activity in real-time. 

In the last decade, many works have reported deployments of monitoring systems using wireless sensor networks (hereinafter WSN), a non-traditional monitoring system, as a new alternative to be considered [17,18]. WSNs are a group of specialized autonomous sensors and actuators with a wireless communications infrastructure, intended to monitor and control physical or environmental conditions at diverse locations, and to cooperatively pass their data to a main location through the network. As they can form networks independently, they can provide environmental information at low operational cost and without periodic maintenance. 

WSN solutions already cover a very broad range of application areas such as medical [19,20,21], environmental [22,23], military [24,25], precision agriculture [26,27,28], animal tracking [29,30], smart spaces [31,32], or surveillance [33,34,35], and research and technology advances continuously expand their application field. With their low power consumption and ease of deployment, WSN are a promising technology when considering volcano monitoring [35,36]. For these reasons, Internet of Things (hereinafter IoT) has acquired great importance in this field. WSN is often a technology used within an IoT system [37]. The IoT enables any item or device with embedded electronics, sensors, actuators, and/or software to connect and exchange data over a network [38]. In the past years, there have been plenty of contributions of the IoT to different application areas such as environmental monitoring [39,40,41,42], smart cities [43,44], smart homes/smart building management [45,46,47], industries [48], healthcare [49], and surveillance [50,51].

Regarding systems based on WSN for volcanic surveillance, most of the research done so far is only focused on the monitoring of seismic or deformation signs, while monitoring the temperature and gas intensity is rarely exposed, although they are important parameters related to volcanic activity [52,53,54,55,56].

This paper’s contributions for researchers in the IoT applications to the volcanic monitoring field, can be summarized as:

1. The first IoT implementation to measure soil temperature in volcanic areas, usually influenced by extreme weather conditions, high temperatures, and the presence of acid gases.
2. Detailed specifications, design considerations, and experimental results for a temperature wireless network. The network suits required and desirable features for volcanic monitoring such as real-time data acquisition, long-range communications, scalability and easy deployment, and also IoT requirements including low cost, low power, long-term service, and high reliability.
3. An extraordinarily energy efficient integration with a specifically design communication protocol.
4. A configuration-free, scalable, compact solution for a fast and effective deployment even in the case of volcanic unrest.

The paper is further organized as follows. An introduction to IoT for volcano monitoring is done in Section 2. A detailed description of the network is documented in Section 3, followed by the measurement results and discussion in Section 4. Conclusions are shown in Section 5.

## 2. IoT for Volcano Monitoring

In the last few years, a lot of new network protocols and devices have been developed. Many of them are well known such as WiFi, Bluetooth, ZigBee, and 2G/3G/4G cellular, but there are also several new emerging networking options such as NB-IoT [57,58], LoRa (long range) [59,60], and Sigfox [61,62]. Depending on the application, factors such as range, data requirements, security, and battery life will determine the most suitable option for communications. Table 1 summarizes the main communication protocols employed for the IoT [63,64,65].

Since volcanic areas are remote and lack easy access to power supply facilities, the chosen communication option must offer both low-power consumption and long-range operation capabilities, that is, low-power wide-area networks (LPWAN) [63,64,66]. A LPWAN is a type of wireless telecommunication wide area network, designed to allow long-range communications at a low bit rate among things operated on a battery. There are a number of competing standards and vendors in the LPWAN space, the most prominent of which include NB-IoT, Sigfox, and LoRa [67].

NB-IoT is a cellular radio access technology set up by the 3rd Generation Partnership Project. It employs quadrature phase shift keying (QPSK) modulation and provides low-power wide-area IoT connectivity in a licensed spectrum and occupies a frequency band width of 200 KHz. Based on the LTE protocol [68], NB-IoT reduces LTE protocol functionalities to the minimum and enhances them as required for IoT applications.

Since this technology operates in licensed bands, NB-IoT has not been considered for the application described in this article, leaving LoRa and Sigfox as the two possible options for the communications.

Sigfox [61,62] is a proprietary ultra-narrowband technology that operates on the 868-MHz frequency band, with a maximum uplink data rate of 100 bps and a maximum packet payload of 12 bytes, allowing to send up to 140 messages per day. It employs the binary phase-shift keying (BPSK), which is a standard radio transmission encoding, taking very narrow chunks of spectrum and changing the carrier phase to encode the data. This allows the receiver to only listen in a tiny slice of spectrum, which mitigates the effect of noise. It requires an inexpensive endpoint radio and a more sophisticated base station to manage the network. Sigfox is a bidirectional communication protocol that shows an asymmetrical connection between the endpoints and the base station. 

LoRa [59] is a physical layer technology that modulates the signals in a sub-GHz Industrial, Scientific, and Medical (ISM) band using a proprietary spread spectrum technique developed and commercialised by the Semtech Corporation [69]. A bidirectional communication is provided by a special chirp spread spectrum (CSS) technique, which spreads a narrowband input signal over a wider channel bandwidth. The resulting signal has noise-like properties, making it harder to detect or jam. The processing gain enables resilience to interference and noise, and provides adaptive data rate capabilities with a maximum payload length of 256 bytes. The communication frequencies that LoRa uses are mainly those that operate with a license-free Industrial, Scientific, and Medical (ISM) radio band, although the technology can operate at any frequency below 1 GHz. This is a key enabler for inexpensive, world-wide deployments and interoperability.

LoRa offers some advantages over Sigfox that are convenient for volcano monitoring, and specifically for the application described in this paper:

- Endpoints and base stations with LoRa-enabled devices are usually more inexpensive than with Sigfox. This is primarily because is possible to use the same radio device for a receiver on the base and at the endpoint stations
- When true bidirectionality is needed, LoRa is likely the better option because of the symmetric link
- LoRa allows to set up and manage the wireless network at a deeper level than Sigfox. The latter provides an API to interact with the radio module, but no configuration is possible
- LoRa has no data limit to be sent per day, and it is not necessary to pay for a renewable subscription plan for each device

For the aforementioned reasons, the communication protocol that best fits the needed requirements is LoRa. 

### 2.1. LoRa for the Thermometers Network

LoRa is a patented [70,71] digital wireless data communication technology developed by Cycleo (Grenoble, France) and acquired by Semtech in 2012. Thanks to the unique chirp modulation, the wireless link can achieve sensitivity up to −148 dBm and up to 168 dB of link budget. It covers a wide range of frequencies, including some license-free ISM bands, and provides data rates in the range of kilobits per second, which is suitable for IoT and Machine-to-Machine applications.

Characteristics of LoRa modulation depend on different parameters [60] configured to tune link performance and energy consumption: transmission power (TP), carrier frequency (CF), bandwidth (BW), coding rate (CR), and spreading factor (SF) (see Appendix A for more detail).

Among the LoRa devices available in the market, the Ra-02 module from Ai-Thinker [72] was chosen to implement communications of the proposed network because of (i) the configuration options, (ii) the price, and (iii) the ease of purchase, given its high availability in the market. This module comes with a Semtech SX1278 [73,74] integrated circuit working on a 433-MHz frequency. This transceiver provides ultra-long range spread spectrum communication and high interference immunity while minimizing current consumption. 

**Figure 1:**
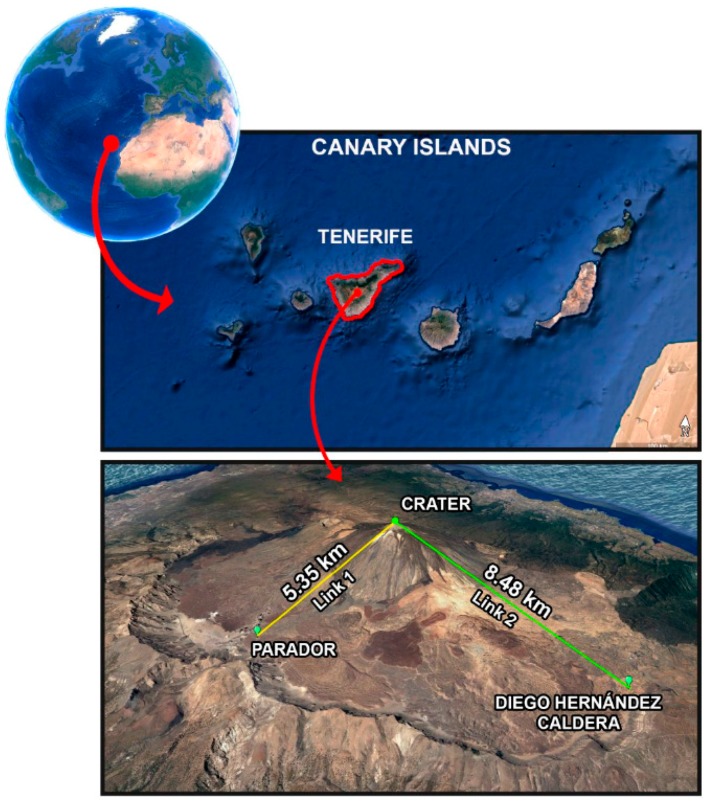
Tested links in Las Cañadas del Teide caldera.

To test the selected module, two communication links were carried out in Las Cañadas del Teide caldera, Tenerife (Figure 1): the first one from the southern rim of the Teide volcano crater to the Parador (5.35 km), and the second one from the northern rim of the crater to Diego Hernández caldera (8.48 km). The crater was chosen because it presents areas of thermal anomalies, the Parador was chosen because there is access to the Internet provided by a station owned by the Spanish National Geographic Institute (IGN), and the point of Diego Hernández caldera was chosen because it allows the largest link within Las Cañadas del Teide caldera. In both links, a 2-dBi dipole antenna in the crater unit and a 9-dBi Yagi antenna in the other two places were used. For a 12-byte message size every 10 s over 15 min, a −123 dBm sensitivity limit value, TP = 17 dBm, CF = 433 MHz, SF = 7, BW = 125 kHz, and CR = 4/5; all messages were received in both links. The received signal strength indicators (RSSI) were −100 dB and −105 dB, respectively: more than 15 dB above the noise level. Therefore, LoRa protocol and the selected module were appropriated to implement long-range links for the proposed network.

## 3. IoT Thermometers Network

**Figure 2:**
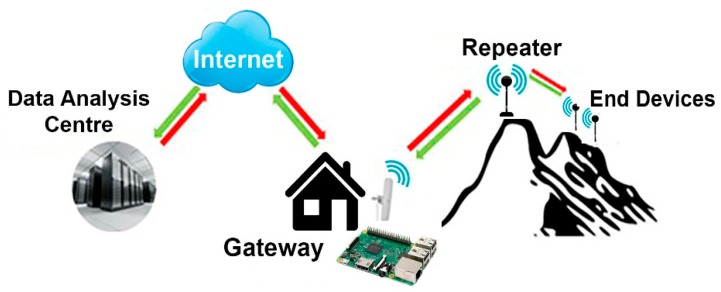
Network operation scheme.

An IoT-based, reliable, robust, low-cost, low-power, and scalable wireless network of thermometers for monitoring the soil temperature in volcanic areas is proposed. This wireless network is composed by three main elements: the gateway, the repeater, and the end devices or thermometers. The network operation can be summarized as follows: The gateway, located in a place with internet access, manages the network and acts as a transparent bridge that relays messages between end devices and a central network server. Every sampling period, it communicates with the repeater to start the temperature measuring process. The repeater will retransmit the order to each end device (its number will depend on the points to be monitored) and wait for the returned answers. Every end device will identify the command and execute the associated task (measure, return RSSI value...), returning to the repeater the required information. The latter will collect packets from each end device, and it will retransmit altogether to the gateway. Data received by the gateway will be processed and stored locally on a SD card, and then, these data will be synchronized to the data analysis center (Figure 2). 

### 3.1. Hardware

#### 3.1.1. The Gateway

For this network, a low-cost LoRa gateway based on a Raspberry Pi [75] was developed. Raspberry Pi has been chosen for different reasons: (i) it can run an operating system based on Linux [76], (ii) it includes multiple communication protocols such as serial peripheral interface (SPI) and TCP/IP, (iii) it has a large development community providing a great amount of code examples and libraries, (iv) its accessibility and hardware price, (v) numerous developments based on Raspberry Pi technology for volcano monitoring show its reliability for its application within this field [15,16,77,78], and (vi) the existence of different great potential units such as Raspberry Shake and Raspberry Boom [79] that could complement the presented network, achieving a complete R-Pi-based IoT monitoring network.

Among Raspberry Pi boards, the model 3B [80] has been chosen because it was the latest product available when the development was carried out. However, the designed gateway is compatible with all the existing Raspberry Pi boards. 

**Figure 3:**
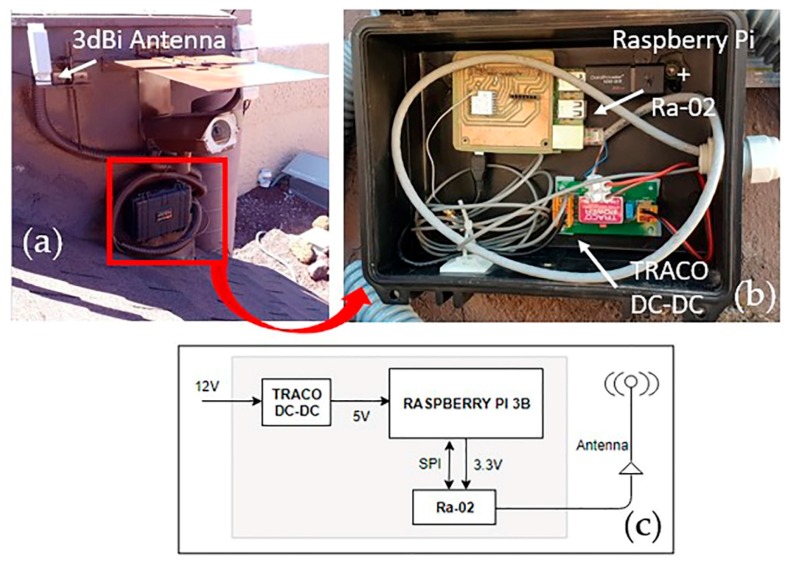
Gateway. Configuration (**a**) and (**b**) and general hardware scheme (**c**).

Raspberry Pi and a Ra-02 LoRa module were used to build the gateway core along with a 3-dBi directional antenna (Figure 3). 

The total consumption of this device was not a key point, since it was thought to be deployed in a place with access to the electrical supply network.

#### 3.1.2. End Devices

**Figure 4:**
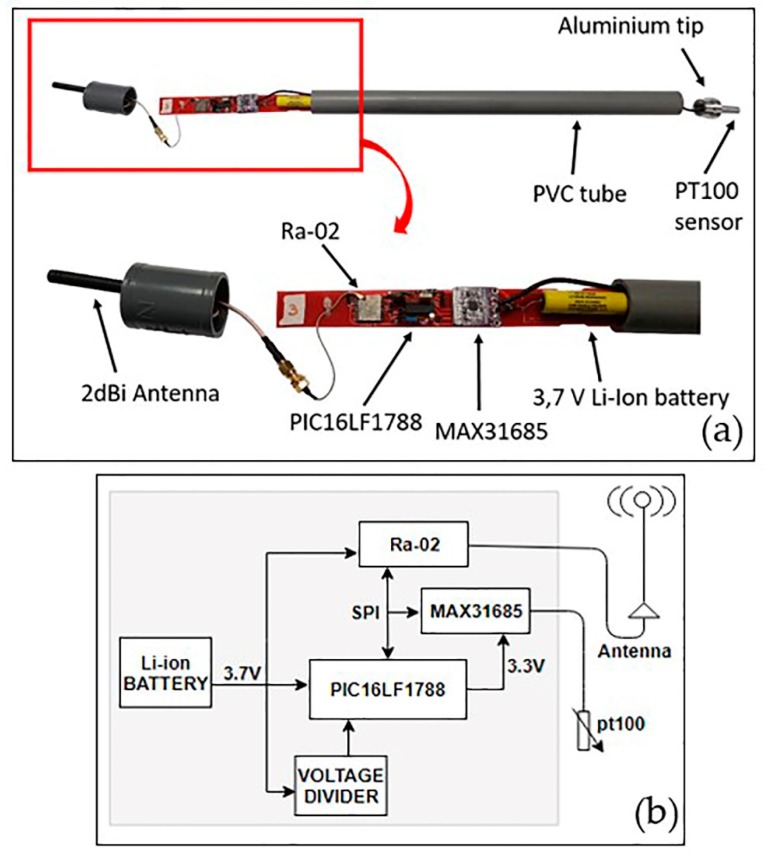
End device. Configuration (**a**) and general hardware scheme (**b**).

Each end device or thermometer is composed by (Figure 4a): (i) a platinum resistance temperature detector (RTD), (ii) a resistance-to-digital converter, (iii) a Ra-02 communications module, and (iv) a control unit (microcontroller) for the system management. A lithium battery was used as power supply. In order to protect these elements from the corrosive volcanic environment and to facilitate the transport and installation, end devices were housed in a PVC tube 60 cm long and with a 32-mm inside diameter. Each tube had at one end a 2-dBi antenna and at the other end an aluminium tip with a specific shape favoring heat distribution. For the temperature measurement, commercial sensors were not used, because they do not resist the corrosion of the acid gases that are characteristic of volcanic environments. Instead, PT100 sensors were placed inside the aluminium tip (Figure 4a), providing protection against acid gases. Thermometers were buried 40 cm deep to measure soil temperature and only the antenna was visible, which was raised 20 cm above the ground. 

##### Control Unit

The microcontroller to be used must meet one crucial requirement: the lowest possible power consumption. According to the minimum features needed, and also taking into account the size and price, different microcontrollers were tested in order to choose the most appropriate: the ATMEGA328 [81] from Microchip, the STM32F103C8T6 [82] from STMicroelectronics, and the PIC18LF25K40 [83] and PIC16LF1788 [84] from Microchip. Each of them was programmed for the temperature acquisition, and then, the total consumption was measured both during operation mode (measuring) and sleep mode (waiting for the next acquisition) using a 3.7-V and 2600-mAh lithium battery. Table 2 shows the results obtained:

Since a system with the longest battery life was desired, and taking into account the results shown in Table 2, the chosen microcontroller was PIC16LF1788 (Table 3).

##### Temperature Measurement

Measurements are made by means of a platinum RTD, PT100B sensor [85], connected to a MAX31685 [86], which is in charge of the conversion of the RTD values into temperature values. The MAX31685 module is an amplifier designed to read the low RTD resistance and automatically compensate it for the resistance of the connecting wires. This module is powered from the GPIO pins of the microcontroller, using SPI protocol for communication between them (Figure 4b).

##### Power Supply

Two different types of lithium batteries were tested in order to choose the best option: a Li-ion rechargeable battery from Samsung [87], and a lithium–thionyl chloride non-rechargeable battery from Keeper [88]. The technical specifications are shown in Table 4.

At first sight, it seemed that the rechargeable battery was the better option, since it had the highest capacity. However, taking into account: (i) the batteries’ operating temperature range, (ii) the expected temperature range in the volcanic area (up to 85 °C), and (iii) the ease of purchase, the two batteries were used in order to test which one was the best option basing on field tests (see Section 4).

The battery was connected to the microcontroller analog to digital converter through a voltage divider in order to monitor its voltage in real-time. In this way, a state of health (SOH) parameter of each thermometer is provided.

#### 3.1.3. Repeater

Volcanic areas usually show a complex orography characterized by deep ravines and a large number of obstacles such as cinder cones. For this reason, a repeater had to be developed in order to extend the gateway coverage, acting as a mid-point between the gateway and the end devices. (Figure 2).

**Figure 5:**
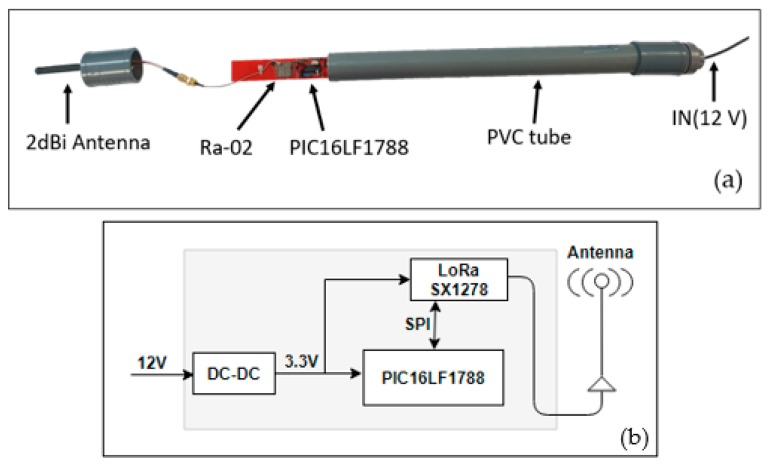
Repeater. Configuration (**a**) and general hardware schema (**b**).

To reduce the development time, the repeater was based on the end device hardware adapted by removing the temperature acquisition module, the RTD, and the MAX31685. Therefore, the repeater is composed by the microcontroller PIC16LF1788, the Ra-02 LoRa module, and a 2-dBi antenna (Figure 5). This device is in receiving mode most of the operation time (see Section 3.2.3), so it has a higher consumption (Table 6) than the end devices. Therefore, it was adapted to be powered by a 12-V battery along with a solar panel.

### 3.2. Software

**Figure 6:**
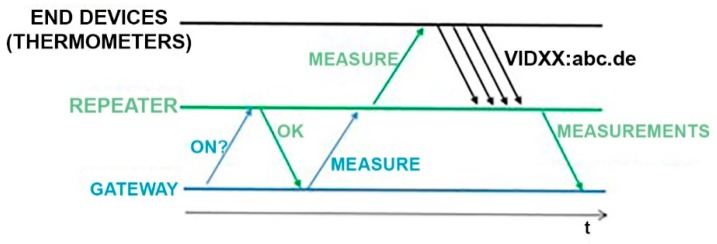
Measurement protocol.

For the network operation, a communication protocol was developed. In this protocol, there is no possibility for a collision among messages, since no element of the network transmits simultaneously to another. For this reason, a single channel is used. When the communication process starts, the gateway and the repeater have both the same configuration parameters (SF and BW), but thermometers are configured with a different SF. The process can be summarized as follows: When an acquisition has to be done, the gateway communicates only with the repeater and test if it is available. If so, the gateway sends the temperature acquisition command to the repeater and keeps waiting a specific time for the returned raw data. Any message that the gateway sends will be received and decoded by the repeater, but not by the thermometers, since they are configured with a different SF (see Appendix A). The repeater, in charge of resending the instruction to the end devices, changes its parameter configuration to match the thermometer configuration so that communication between them is possible. The repeater sends them the temperature acquisition command and keeps waiting for their answers. Thermometers receive the command, perform the corresponding tasks, and send the information back to the repeater in their turns (thermometer 1 waits one second and transmits, thermometer 2 waits two seconds and transmits, and so on). The repeater receives the raw data, returns to initial parameter settings, and transmits the information to the gateway, who stores it (Figure 6).

In order to manage the network properly, a software development had to be made. The set of programs and scripts developed involves several technologies and program languages such as: Python, C, and HTML, running each one in different devices in the network. Some scripts are executed using Linux cron, which is a background process manager that is able to run processes at regular intervals, which can be scheduled.

The compiled programs and written scripts are listed in Table 5.

#### 3.2.1. Gateway Software

**Figure 7:**
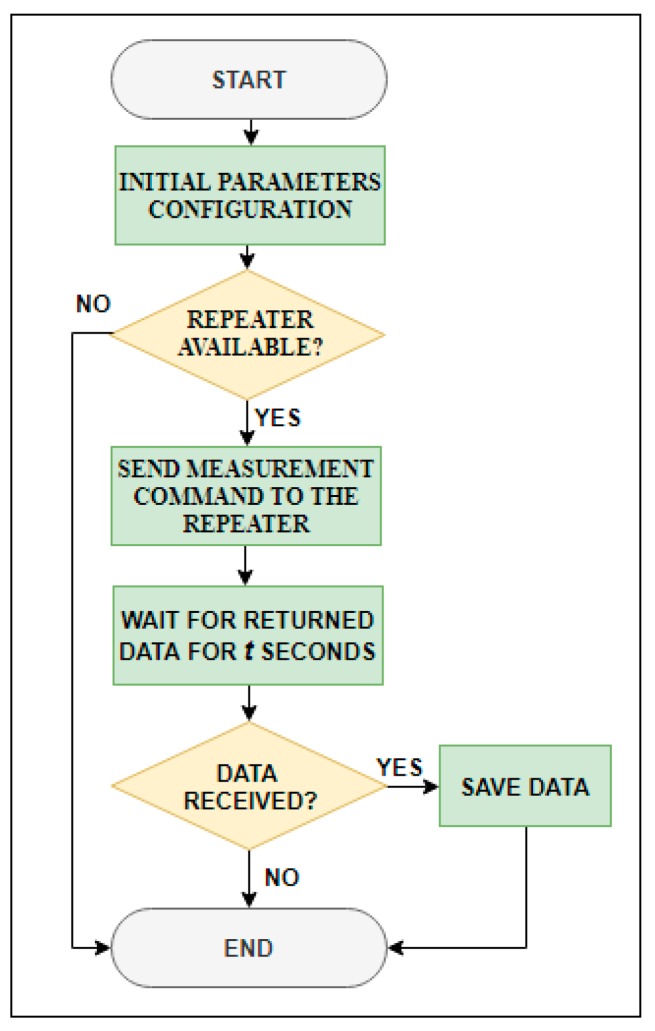
Gateway flow chart.

As mentioned, the gateway is in charge of managing the wireless network running the master_main.py script (Figure 7). First, it tests if the repeater is available. If so, the gateway sends the temperature acquisition command to the repeater, which resends the instruction to the end devices. If the repeater is not available, the process ends until the next acquisition. 

The gateway waits a specific time for the returned raw data. When data are received, the calibration curve of each temperature sensor (included in master_main.py) is applied, and then they are time stamped and stored in a USB flash drive. If a thermometer does not respond, its stored value will be “Nan”. The storing memory has a structure of nested folders as follows: /thermometer_data/year/month, in which files are stored on a daily basis.

Using the sync.sh script, running by default every 10 min, stored data is fully mirrored to a remote computer (data center). Data transfer is performed using rsync Linux utility [89], which minimizes the volume of data transmitted, since it employs a delta encoding algorithm that only stores bytes that have been modified since the previous version of the file. 

A master_onesample.py script operates in the same way as master_main.py, but when sending the measurement command to the end devices, they will remain in receive mode instead of going to sleep mode. This slight difference allows using this script during the installation and/or when it is desired to obtain temperature values at specific times. If the end devices’ consumption and power supply are not a problem, this script can be used to manage the network.

The last script running in the gateway is Rssi.py, which is only executed under the user’s request. This script allows knowing the RSSI level of every device in the wireless network. First, it tests if the repeater is available, and if so, it sends the RSSI request for a certain device of the network, repeater, or end device, and waits during a fixed time period for the requested information. If the repeater is asked, the RSSI level is the power level of the radio signal received in the gateway emitted by the repeater. If an end device is asked, the RSSI level is the power level of the radio signal received in the repeater emitted by the end device.

#### 3.2.2. Repeater Software

**Figure 8:**
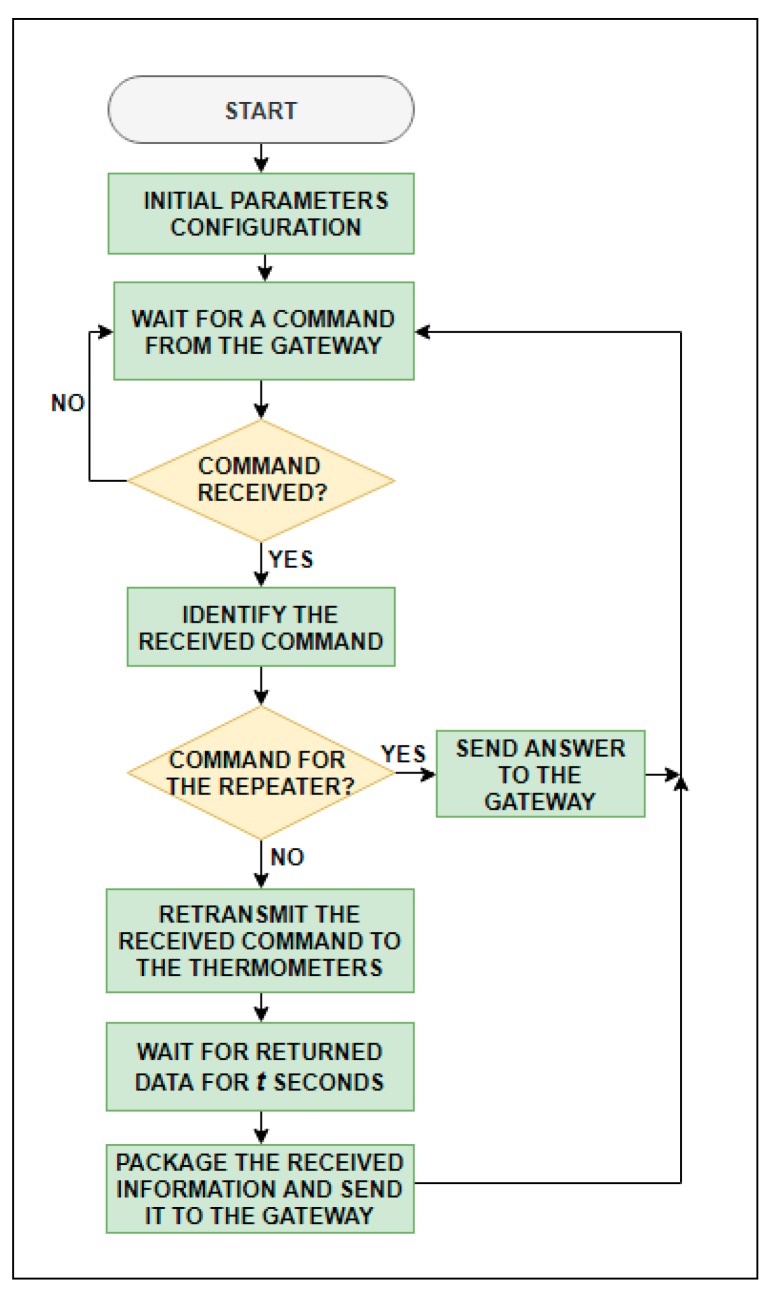
Repeater flow chart.

The Repeater.cpp program manages the repeater operation (Figure 8). The default status is receiving mode (Section 3.2.3), meaning that it is always waiting for a message from the gateway. Depending on the command received, two different actions are executed. On the one hand, if the final recipient is an end device, the repeater relays the message and keeps waiting for the answer during a fixed time period, which depends on the number of end devices that compose the network. Then, it packages all the information received from the end devices, adds some extra information such as each end device RSSI, sends it to the gateway, and returns then to receive mode. On the other hand, if the final recipient is the repeater itself, it automatically answers the requested information and returns to receive mode.

#### 3.2.3. End Devices Software

**Figure 9:**
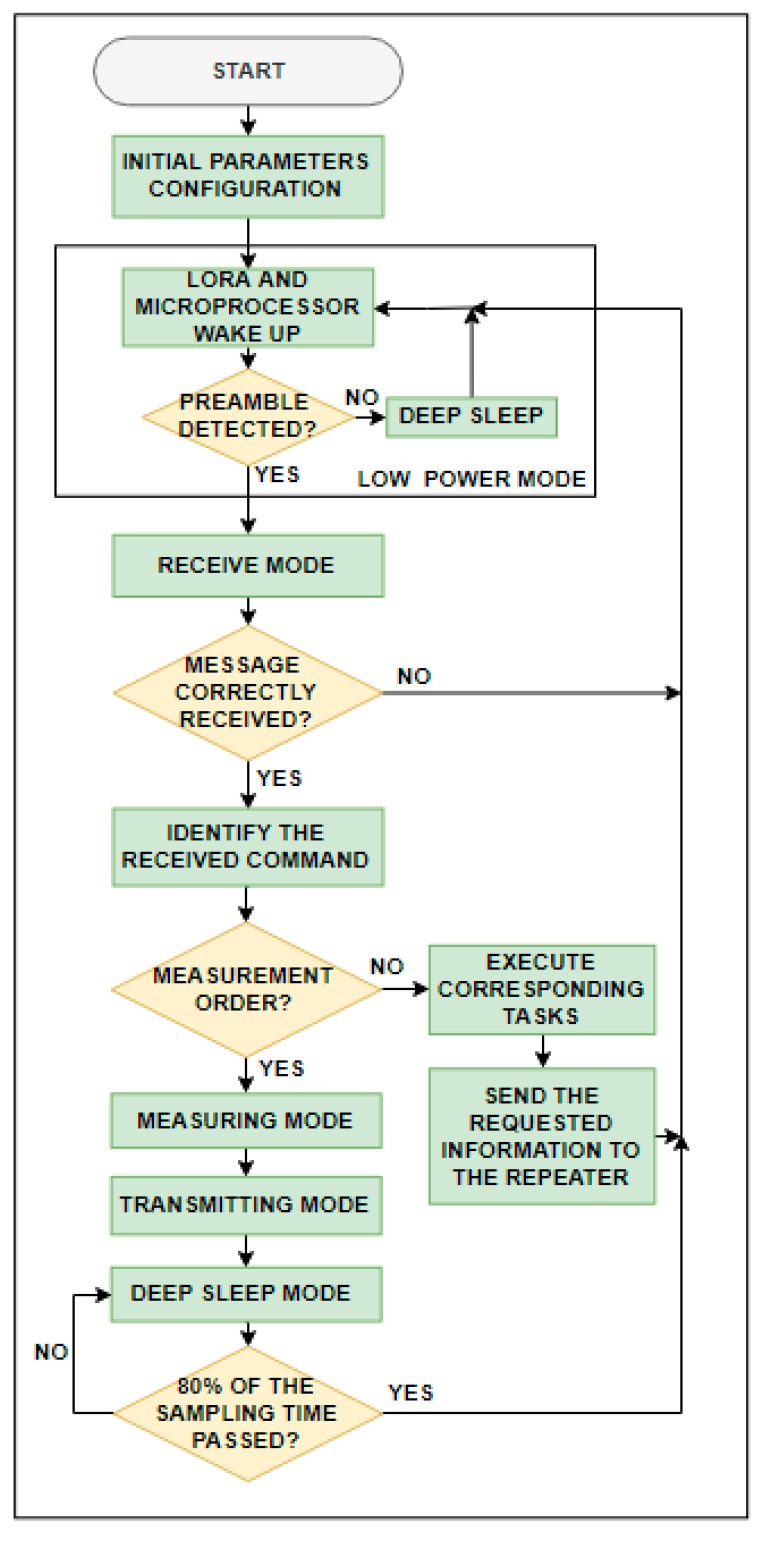
End devices flow chart.

The end_device.cpp program manages the end device operation (Figure 9) by means of five different states:

1. Deep sleep mode

The microcontroller is in deep sleep mode. The rest of the elements are off. In this mode, the end device shows the lowest power consumption (Table 6).

2. Low power mode

The end device is waiting to detect any preamble in the air. In this mode, the following routine is repeated: from deep sleep mode the microprocessor and the LoRa module wake up every certain programmed time by means of the microprocessor timer to check if any preamble is available. If so, the end device changes to receive mode. 

3. Receiving mode

The end device enters in this mode when it detects a preamble in the air, waiting until it receives the complete message. If the message has been received correctly, the end device identifies it and executes the corresponding task. If the received command is to measure the temperature, the end device turns to measuring mode. If the received command is an RSSI request, the end device changes directly to transmitting mode and sends the corresponding information. 

4. Measuring mode

In this mode, the end device performs the temperature measurement by making 10 consecutive measurements in one second and later calculates a mean. Then, it waits to send it to the repeater (transmitting mode) in its corresponding turn (turn time in seconds is equal to the identification number of each end device). Finally, the end device turns to deep sleep mode during 80% of the sampling time in order to save as much energy as possible.

5. Transmitting mode

Data are sent to the repeater with the following format: VIDXX: abc.de,f.gh, where ID is the identifier of the end device, abc.de is the data requested, and, f.gh is the battery voltage.

#### 3.2.4. Server Software

Since the gateway synchronizes data to the data analysis center every 10 min, is possible to display the measurements in real-time. For this purpose, a web page interface was developed. Users can check the system operation and view data in real-time. The website is built by means of join.py and LoRa_represent.py python scripts, which are executed every 10 minutes using cron of Linux.

**Figure 10:**
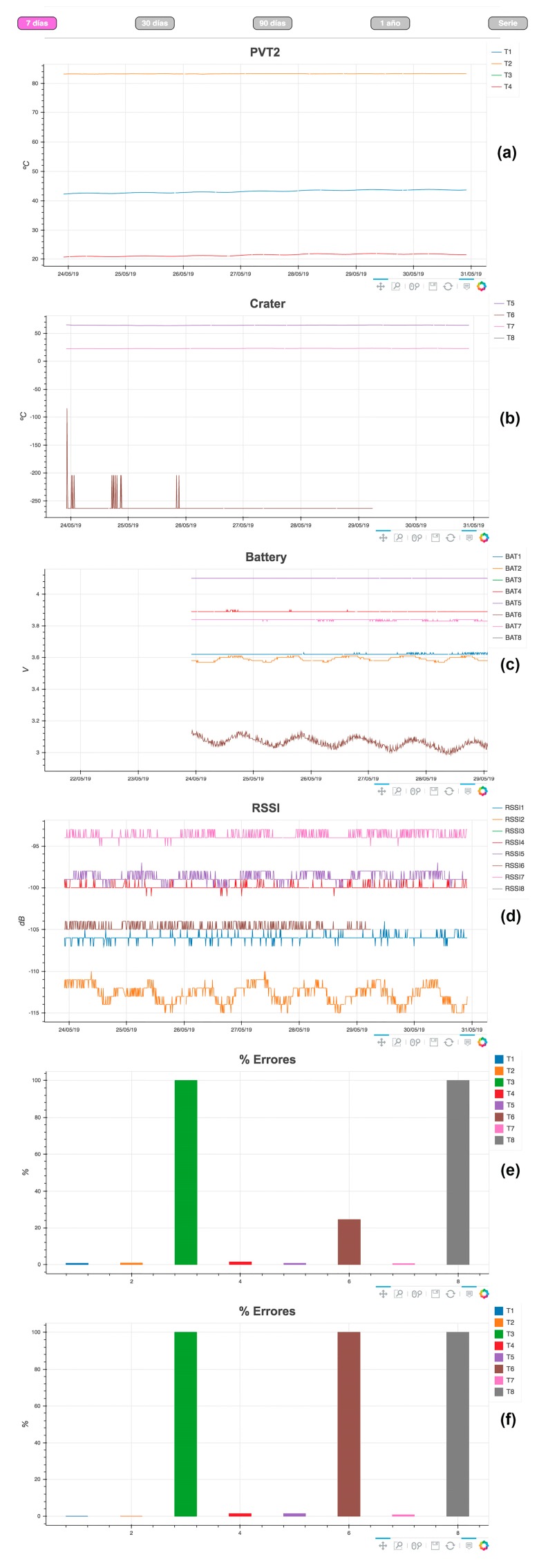
Webpage. (**a**) Temperature values measured by thermometers in area A; (**b**) Temperature values measured by thermometers in area B; (**c**) Battery level of each thermometer; (**d**) RSSI level of each thermometer measured in the repeater; (**e**) Percentage of error of each thermometer in the last 24 h; (**f**) Percentage error for the selected time period.

The join.py script joins in a single file all the files stored in the nested folders in the database. Then, the LoRa_represent.py script reads the file generated by join.py and plot data in HTML files by means of the Bokeh library [40] and P5.JS [90]. As a result, five HTML files are obtained with different time periods: 7days.html, 30days.html, 90days.html, 1year.html, and series.html. Each html file has its own tab in the web page (Figure 10) where users can select the desired time period. Each html file is organized in five plots as follows: temperature values recorded, battery level of each thermometer, RSSI level, measurement percentage of error for each thermometer in the last 24 h (only in 7.html file), and measurement percentage of error for each thermometer along the time period displayed (Figure 10).

### 3.3. Power Consumption

Since end devices are powered only by batteries, power consumption has to be as low as possible. In order to estimate its power consumption in the different modes (see Section 3.2.3), one end device with a 10-minute sampling period (the fastest possible) powered by a 2200-mAh battery was employed. The results are shown in Table 6.

Basing on these results and assuming an 80% maximum discharge level for the battery, a battery life of 2.14 years was theoretically estimated for the end devices when using a 2200-mAh battery.

### 3.4. Price

Table 7 shows the price of each element of the network.

## 4. Results and Discussion

### 4.1. Site Selection

**Figure 11:**
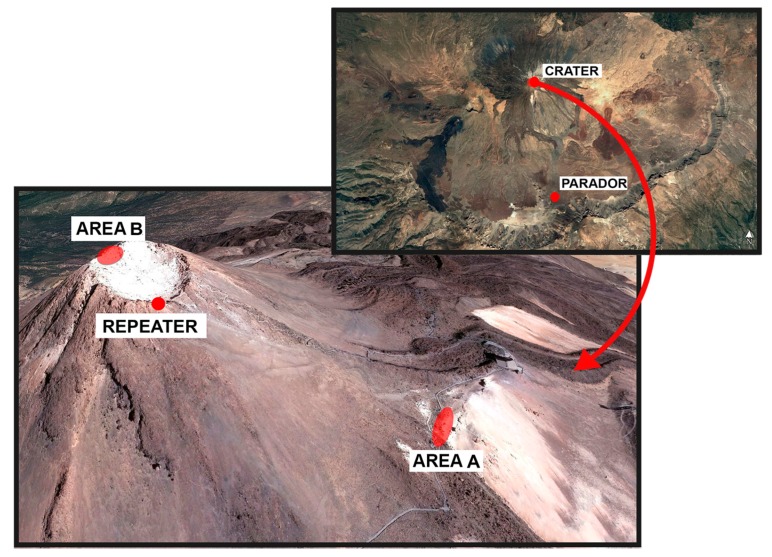
Wireless network configuration installed in Las Cañadas del Teide, Tenerife. Devices were deployed as follows: the gateway in the Parador, the repeater at the southern rim of Teide crater, and the eight thermometers on the summit part of Teide volcano (areas A and B).

The network described in this paper was installed on 20 November 2018 in Las Cañadas del Teide in Tenerife, Canary Islands. The gateway was deployed in the Parador, whilst the rest of the elements (repeater and eight thermometers) were placed on the summit part of Teide volcano, areas A and B (Figure 11), where the main thermal anomalies are located. 

In the Parador, the gateway was installed taking advantage of the location of a video camera that is integrated into the Canary Islands volcano monitoring system belonging to the Spanish Instituto Geográfico Nacional (Figure 3a).

The repeater was placed ensuring a proper line of sight to the Parador in order to improve the signal strength of the gateway–repeater link (Figure 11 and link 1 in Figure 1). 

The locations of the thermometers were carefully selected based on data collected by the MultiTeide project [91] (Villasante personal communication). Since October 2016, every three months, temperature measurements at 40-cm deep were carried out in the main thermal anomaly areas on the summit part of Teide volcano. Two of them are A and B areas (Figure 11). Area A is located at the southeast zone in the upper part of the Teide edifice, and area B is located inside the Teide crater itself.

**Figure 12:**
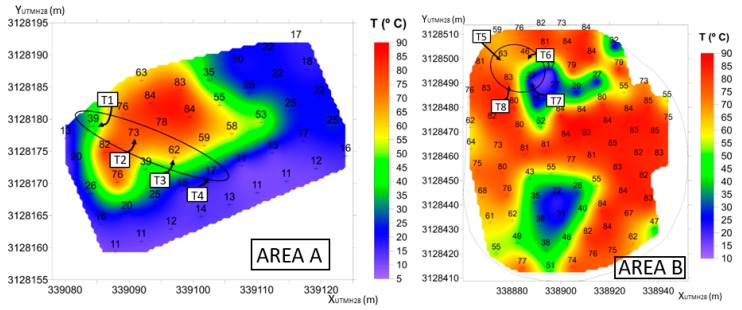
Temperature maps of the areas A and B provided by MultiTeide campaigns (not published). The location of each thermometer within both areas is highlighted.

Three main criteria were taken into account to select the final configuration of the eight thermometers, equally distributed between the two areas: (i) points that show higher temperature variations over time, (ii) points with the highest and lowest temperature values, and (iii) monitoring zones that show less stability throughout the year. Considering this, the final thermometer distribution selected is displayed in Figure 12. Four thermometers were place in area A (T1–T4) forming a linear profile, and the other four (T5–T8) were deployed in area B, forming a square.

It is necessary to point out that the network is scalable, being possible to expand or reduce the number of end devices even after deploying it only by modifying a single parameter in the gateway configuration.

### 4.2. Network Configuration

Taking into account the final network layout (Figure 11), and to achieve an optimal operation, the lowest possible power consumption and a sufficient sensitivity to accomplish the detection, the values selected for the network configuration parameters are described below. There are some LoRa parameters that remained fixed such as CF, TP, BW, and CR, and others such as SF or the preamble length that are adapted depending on which elements are communicating:

- CF = 433 MHz, the license-free band (Europe) with the greatest range
- TP = 10 dBm (10 mW effectiv radiated power), the maximum transmission power in order to ensure that messages reach the receiver
- CR = 4/5 because there is not too much interference, thus guaranteeing a shorter time on air (see Section 2.1)
- BW = 125 kHz to achieve sufficient sensitivity for the detection
- For SF, the lowest possible value that allows a correct operation of the network has been chosen. A low SF allows for less time on air and therefore less consumption (see Section 2.1). There are several SF configurations within the network: SF = 7 for the repeater–thermometer link, and SF = 8 for the gateway–repeater link because it is longer than the other
- The preamble length is adapted to two different situations. As previously mentioned, at least 4.25 symbols are needed for the detection. In cases where the receiver is not in deep sleep mode, a short preamble will be enough, as in the case of the gateway–repeater communication in both directions, and also when the thermometers talk to the repeater (see Section 3.2.1 and 3.2.2). In this case, the chosen preamble length is 12 symbols. When the repeater communicates with the thermometers, the latest are in low power mode (see Section 3.2.3). Taking into account that one cycle of the low power mode lasts 1000 milliseconds (980 ms in deep sleep mode and 20 ms listening) and the following equation [74]:
  (1) $$\mathrm{Tsymbol}=\;\frac{\;2^{SF}}{BW}$$
  where Tsymbol stands for the symbol duration in seconds or the time taken to send 2^SF^chips and BW stands for the bandwidth. For SF = 7 and BW = 125 kHz, Tsymbol = 1.024 milliseconds. Therefore, a preamble length of 1100 symbols has been chosen so that even in the most unfavorable case (preamble transmission starts when the thermometer goes to deep sleep mode), the detection occurs.

### 4.3. Data Records

The sampling frequency is 10 min to ensure a proper recording of soil temperature, although different time intervals can be programmed via Secure Shell (SSH) and modifying the corresponding file.

The temperature wireless network has been operating for over three months, allowing the verification of the robustness of both the network operation and each individual device, and the quality of temperature recordings. Up to the date of sending this article, it is still continuing to work. Table 8 summarizes the data collected by the network since it was installed.

**Figure 13:**
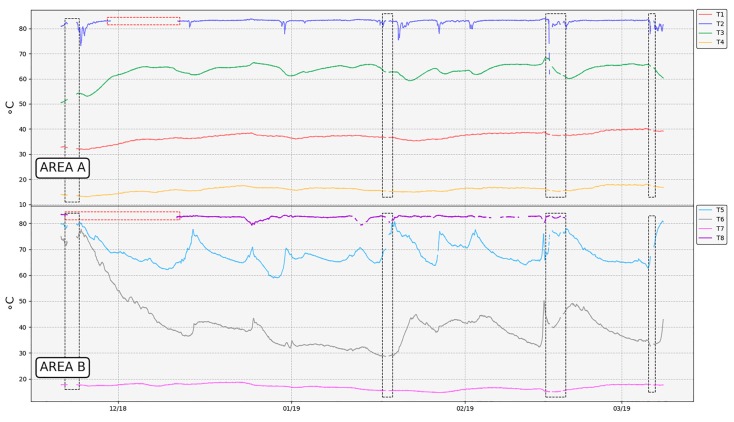
Temperature records. Grey dashed rectangles point out temporally interruptions in communications due to adverse weather conditions. Red dashed rectangles highlight malfunctions in thermometers T2 and T8.

Under normal conditions, the operation of the network is quite stable, and the temperature measurements of each thermometer match the expected values according to MultiTeide campaigns (Table 8, Figure 12 and Figure 13). Variations in the temperature values are associated with changes in meteorological conditions and/or volcanic activity [92]. However, during this period of time, there were some issues that affected the normal function of the network.

After the network deployment, there were some periods when the weather conditions in Las Cañadas del Teide worsened notably, especially in the Teide crater, where rains and snowfalls took place and a layer of thick clouds was permanently established. Therefore, the network robustness was able to be tested under adverse weather conditions. Due to the layer of snow that covered both the repeater and the thermometers, the RSSI of all these elements decreased. In addition, the layer of clouds that settled frequently on the summit part of Teide volcano interrupted the gateway–repeater link. This was mainly because the gateway antenna has low gain, so the gateway–repeater link was the weaker one when extreme adverse weather conditions took place (therefore, one possible improvement would be to use a higher gain antenna).

**Figure 14:**
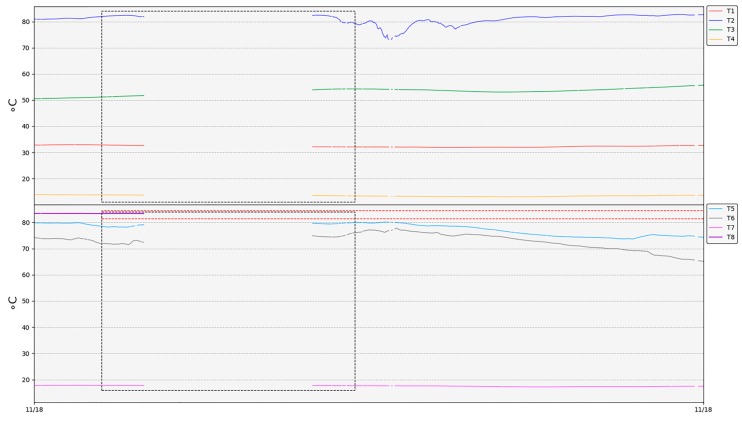
Detail of the temperature record when interruptions in the network operation (grey dashed rectangle) take place due to adverse weather conditions.

Consequently, temperature measurements were not acquired (grey dashed rectangles in Figure 13 and Figure 14) during these periods. Once the weather conditions became more favorable, the snow melted and the network returned to operation again (Figure 13 and Figure 14).

When a transmission error occurred, no temperature registration value was available for that thermometer in that sample, which translated into a gap (Figure 13).

**Figure 15:**
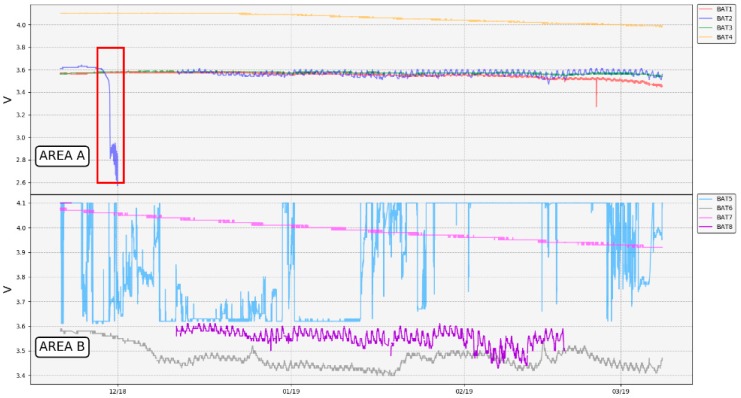
Battery records. The red rectangle highlights the T2 battery drain due to overheating.

**Figure 16:**
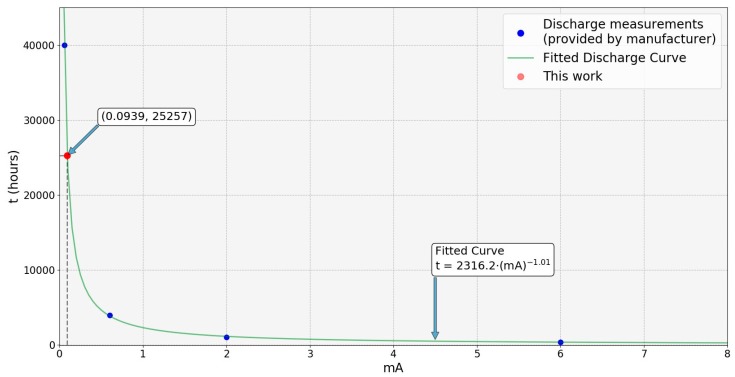
Fitted curve for battery life versus current consumption. Blue points highlight values obtained from the discharge curves provided by the manufacturer. The red point highlight the value corresponding to this work.

Figure 15 and Table 8 display the battery records of each thermometer. T1, T2, T3, T6, and T8 thermometers are powered by non-rechargeable 3.6-V batteries, and thermometers T4, T5, and T7 are powered by Li-ion rechargeable 3.7-V batteries. Except thermometer T5, whose recorded values where erroneous (Figure 16, blue line in area B) due to a hardware problem with the voltage divider that allows to measure the value of the battery, it can be seen that most of the curves displayed are fairly constant with a very slow battery discharge. 

Besides the commented transmission issues between the gateway and the repeater, Figure 15 also shows problems related to batteries and the temperature operation.

When designing the thermometer, it was assumed that the PVC tube would be able to act as a protection and insulator of the electronics against the corrosive gases and the high soil temperature. It was also thought that the part of the tube that was not buried would act as heat sink, since it was in contact with the air. While thermometers were tested in the laboratory, no problems related to batteries arose, since temperature values measured did not exceed 30 °C. However, once the network was deployed in the selected area, it was found that measures used to dissipate the heat inside the tube did not work as expected, and the temperature inside the tube probably exceeded what was recommended for the battery (Table 4). Analyzing capacity versus current curves depending on the temperature of operation provided by the manufacturer [88], it can be seen that the capacity of the battery is greatly reduced as the temperature increased, even though consumption is minimal, as in the case of thermometers. As a result, some batteries were completely drained out, as can be observed in Figure 15 for thermometer T2 (red rectangle) and consequently, the thermometers associated stopped working (red dashed rectangles in Figure 13).

Regarding thermometer T8, on 8 November 2018, it was checked in situ that the battery was completely discharged. The most plausible explanation was that the battery drained out due to inefficient heat dissipation inside the T8 tube. The discharge record was not acquired as in T2, since during the first days of the network operation, the weather conditions did not allow proper communication among the network devices.

The overheating problem affected not only the integrity of the batteries, but also the capacity, and therefore, the battery life. Taking the current values and the time in hours from the battery discharge curve provided by the manufacturer [88], a fitted curve has been drawn (Figure 16).

Starting from the values shown in Section 3.3, the average consumption of a thermometer is 0.0939 mA. Taking this value and the discharge fitted curve shown above, the estimated battery-life value assuming an 80% maximum discharge level is 2.34 years, which is a very similar value to the one theoretically calculated (Section 3.3). However, taking the capacity versus current curves depending on the temperature of operation provided by the manufacturer [88], for the temperature ranges in which the network works, the capacity of the battery is reduced to 1400 mAh, and thus, 1.46 years would be a more accurate value for the battery life of the device.

Analyzing the overheating batteries issue, it was necessary to ensure that they did not exceed the operating temperature range as far as possible. First of all, drained batteries were replaced by 3.7-V non-rechargeable batteries, whose range of operation was more suitable for this application (Table 4). Then, in order to improve the batteries’ cooling, they were changed in thermometers T2 and T8 from their initial position (Figure 4) to the top of the PVC tube. In addition, a thermal insulator was placed at half height inside the tube to reduce the heat transmission from its bottom. After these changes, T8 was poorly sealed, allowing water to filter inside the PVC tube, deteriorating the electronics little by little. This thermometer began to show a malfunction two months later, failing to acquire temperature values and showing some communication issues (which increased the error percentage in the T8 packets reception, Figure 18), until it finally stopped working. With regard to the rest of the network, it became fully operational again, and since then, data has been recorded without problems thanks to favorable weather conditions (Figure 13).

**Figure 17:**
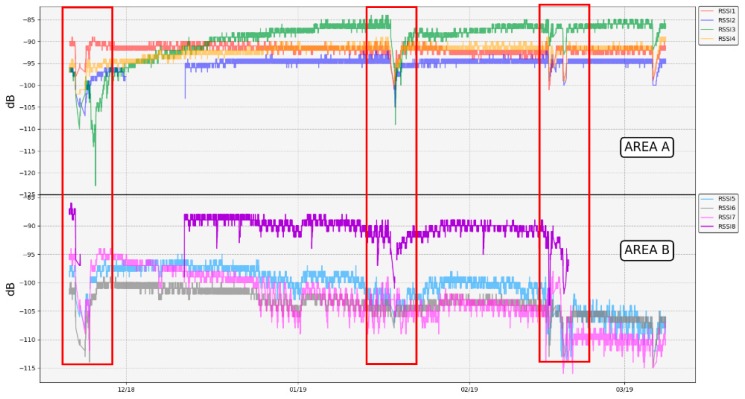
RSSI levels of each thermometer in areas A and B measured at the repeater side. Red rectangles point out periods in which the reception of packets in the repeater gets worse due to adverse weather conditions.

Figure 17 and Table 8 show the RSSI levels of each thermometer measured at the repeater side. In general, RSSI levels remained quite stable and above the sensitivity limit of −123 dBm according to LoRa parameters of the repeater–thermometer link (SF = 7 and BW = 125 kHz). However, Figure 16 displays different periods in which, due to the worsening of the weather conditions, a drop in the RSSI levels associated with a decrease in signal strength takes place. If high signal attenuations occur, these can lead to interruptions in the operation of the network (grey dashed rectangles in Figure 13 and Figure 14). As these conditions gradually improve (possible ice sheets melted, visibility between devices was greater, etc.), the network recovers and continues with normal operation (red rectangles in Figure 17).

An estimation of the error in the reception of each thermometer packages was done computing the received and theoretical received packages ratio as follows:

(2) $$\mathrm{ERROR}=\;1-\frac{\;NRM}{TNM}\cdot 100\%$$

**Figure 18:**
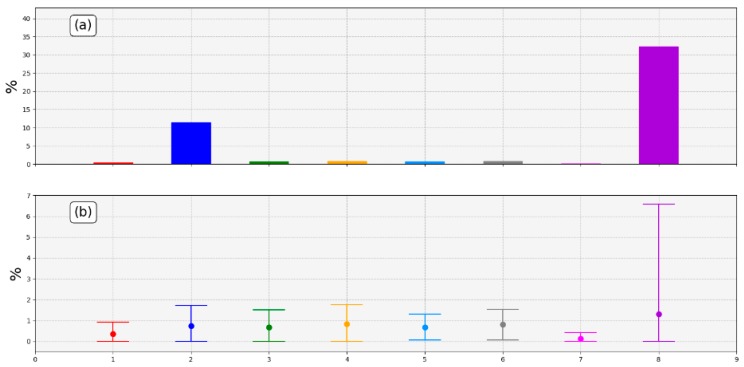
Estimation of errors. (**a**) Percentages of total error in transmitted packets applying Equation (1); (**b**) 24-hour mean transmission error ± standard deviation.

Where NRM stands for the Number of Recorded Measurements in a specific time period, and TNM stands for the Theoretical Number of Measurements in the same period. Equation (2) was applied to the period of time in which all the thermometers have been working, and the results were plotted in Figure 18.

T2 and T8 percentages of error in 24 h are the highest ones due to the problem with batteries already mentioned. The percentages of transmission errors of the remaining thermometers were quite low (below 2% in 24 h). A percentage of error so low allows to maintain a suitable temperature sampling when the weather conditions are sufficiently favorable, revealing the robustness of the designed network. 

Differences between thermometers were due to the different characteristics of the sites in which they were settled, the weather conditions, the distance to the repeater, whether there was direct vision with the repeater or not, and the incidence of the sun (related to the melting of the ice that could form). 

## 5. Conclusions

This article describes the development of the first reliable, robust, low-power, and low-cost scalable wireless network based on IoT that can be used for monitoring thermal anomalies in volcanic areas in real-time. Taking advantage of LoRa communication protocol, the designed network of portable thermometers (eight in total) allows continuously monitoring the temperature values on different points in the desired areas, showing the acquired values to remote users through a user-friendly web dashboard. Besides the temperature values, users can also visualize parameters related to the network operation such as RSSI, the battery levels of each thermometer, and the percentage of error in the transmission of the packages.

The main advantage of the network presented here is its extremely low power consumption, which ensures a long battery life. A theoretical continuous operation of 2.14 years (Section 3.3 and 4.3) was estimated for each thermometer powered by a 3.7-V single battery and transmitting data every 10 min. This result assures both a proper sampling for soil temperature and long time period with no maintenance, which is vitally important in volcano monitoring, since these areas are usually remote and inaccessible. 

A low cost was achieved since the total price for the network is 384.75 €, with the gateway being the most expensive device (Table 7). Each thermometer had an estimated cost of 37.01 € and they were designed with a suitable shape to facilitate the transport and installation. In this way, a reliable network can be quickly deployed with the proper number of thermometers and with an affordable price. In addition, and if it is needed, after the initial deployment, the network can be easily enlarged by adding new thermometers simply by modifying one parameter on the gateway side.

The network was installed on 20 November 2018 in Las Cañadas del Teide in Tenerife (Spain) and continues to work. The temperature values recorded (Figure 13 and Table 8) matched with previous data reported by the MultiTeide project. Thus, proper temperature monitoring is guaranteed by the developed wireless network.

Regarding the RSSI levels (Figure 17), LoRa has showed a very good performance, confirming that it was the right choice among different transmission options. When weather conditions were not adverse, RSSI values remained higher than the sensitivity level in all thermometers. As a consequence, the network was operable the majority of the time, reaching a very low percentage error in the packages transmission: less than 2% in 24 h (Figure 18).

Therefore, all these results confirm that the developed wireless network designed for monitoring the soil temperature in volcanic areas has accomplished the initial requirements of low power, low cost, scalable network, robust operation, and real-time communication. With minimum changes and improvements, this wireless network could be deployed in other volcanic areas to strengthen the volcano monitoring programs. 

## Figures and Tables

**Table 1:** Internet of Things (IoT) main communication technologies.

	Frequency Bands (MHz) ^1^	Nominal Range	Data Rates ^1^
NFC	13.56	10 cm	100–424 kbps
Bluetooth	2400	0.5–100 m	1 Mbps
ZigBee	2400	10–100 m	250 kbps
WiFi	2400/5000	50–100 m	150–250 Mbps (typical)
Cellular	900/1800/ 1900/2100	35 km max for GSM, 200 km max for HSPA	35–170 kbps (GPRS) 120–384 kbps (EDGE) 384 kbps–2 Mbps (UMTS) 600 kbps–10 Mbps (HSPA) 3–10 Mbps (LTE)
NB-IoT	Licensed LTE frequency bands	10 km (rural), 1 km (urban)	20/200 kbps ^2^
Sigfox	868	30–50 km (rural), 3–10 km (urban)	100/600bps ^2^
LoRa	433/868	15–20 km (rural), 2–5 km (urban)	0.3–50 kbps

^1^ European Union values, ^2^ Uplink and downlink data rates.

**Table 2:** Microcontrollers’ power consumption.

Microcontroller	Sleep (µW)	ON (mW)
ATMEGA328	16.28	89.54
STM32F103C8T6	12.58	190.92
PIC18LF25K40	45.88	69.93
PIC16LF1788	2.96	70.3

**Table 3:** The PIC16LF1788 features.

PIC16LF1788 Features	
Program Memory Size	28 KB (Flash)
CPU Speed/Internal oscillator	8 MIPS/ 32 MHz
SRAM	2048 Bytes
Data EEPROM	256 Bytes
Digital Communication Peripherals	1-UART, 1-SPI, 1-I2C1MSSP(SPI/I2C)
Timers	2 × 8-bit 1 × 16-bit
ADC Input	11 ch, 12-bit
Temperature Range	−40 to 125 °C
Operating Voltage Range	1.8 to 5.5 V
Low Power	Yes

**Table 4:** Battery specifications.

	Li-Ion Rechargeable Battery	Lithium Thionyl Chloride Non-Rechargeable Battery
Type	18650	AA
Nominal Voltage	3.7 V	3.6 V
Charging Voltage	4.2 ±0.05 V	-
Discharge Cut-Off Voltage	2.75 V	2.0 V
Capacity	2600 mAh	2400 mAh
Operating Temperature Range	−20 → +60 °C	−55 → +85 °C

**Table 5:** Network software. RSSI: received signal strength indicators.

Device	Software/Script	Execution	Programming Language	Purpose
Gateway	master_main.py	Every 10 min (default)	Python	Asks the end devices for temperature value
	synch.sh	Every 10 min	Linux Bash shell	Synchronizes data between gateway and server
	Rssi.py	At user’s will	Python	Asks for the RSSI of any device in the network
	master_1sample.py	At user’s will	Python	Asks the end devices for temperature value
Repeater	Repeater.cpp	Continuous	C	Relays messages between the gateway and the end devices
End device	End_device.cpp	Continuous	C	Temperature measurement
Data Analysis Center	join.py	Every 10 min	Python	Joins all temperature daily files in a single one
	LoRa_represent.py	Every 10 min	Python	Takes the joined file and creates a graphical representation in HTML

**Table 6:** Total power consumption.

Mode		Power Consumption (mA)	Time Percent Per Hour
Transmitting		70	0.0075%
Receive mode		12.2	0.2%
Deep Sleep mode		0.0035	80%
Low Power mode	Sleep	0.0035	19.0699%
	Receive	12.2	0.3891%
Measuring		4	0.3333%

**Table 7:** Total price.

END DEVICE (THERMOMETER) COMPONENTS	Price (€)
PT100 sensor	5.50
MAX31685	4.11
PIC16LF1788	6.04
External structure (PVC pipe + aluminum tip)	1.37
Ra-02	3.00
Antenna	5.45
Lithium battery	9.54
Wires, cases and components	2.00
**Total price of one end device**	**37.01**
**GATEWAY COMPONENTS**	
Raspberry Pi 3B	35.66
Ra-02	3.00
Antenna	22.61
**Total price of the gateway**	**61.27**
**REPEATER COMPONENTS**	
PIC16LF1788	6.04
External structure (PVC pipe + aluminum tip)	1.37
Ra-02	3.00
Antenna	5.45
Lithium battery	9.54
Wires, cases, and components	2.00
**Total price of the repeater**	**27.40**

**Table 8:** Descriptive statistics of the data recorded by the network.

	Samples		T (°C)	RSSI (dBm)	BAT (V)
**T1**	14522	MIN	31.87	–101	3.27
		MED	36.95	–92	3.55
		MAX	40.18	–89	3.58
**T2**	13005	MIN	61.91	–107	2.57
		MED	82.82	–95	3.56
		MAX	83.88	–92	3.64
**T3**	14480	MIN	50.52	–123	3.54
		MED	63.17	–89	3.57
		MAX	68.31	–84	3.59
**T4**	14445	MIN	13.04	–102	3.98
		MED	15.95	–92	4.06
		MAX	18.00	–89	4.10
**T5**	14466	MIN	58.89	–114	3.61
		MED	68.70	–101	3.87
		MAX	81.09	–95	4.10
**T6**	14445	MIN	28.68	–115	3.40
		MED	41.08	–103	3.47
		MAX	77.77	–99	3.59
**T7**	14551	MIN	14.75	–116	3.92
		MED	16.90	–103	3.99
		MAX	18.75	–94	4.08
**T8**	9565	MIN	79.29	–104	3.43
		MED	82.43	–90	3.56
		MAX	83.41	–86	4.10

## Appendix A

The LoRa solution can be labeled CDMA (Code Division Multiple Access), using different spreading factors and coding rates (see Appendix A) to multiplex signals on a single frequency. This not only increases the network capacity, but also allows for the dynamic adaptation of device data rates.

Spreading a narrowband signal over a wide band results in less efficient use of the spectrum. However, this problem is typically overcome by the use of multiple orthogonal sequences. As long as multiple end devices use different channels and/or orthogonal sequences, everything can be decoded concurrently, resulting in a higher overall network capacity.

The different parameters that define the characteristics of LoRa modulation are described below:

- Transmission Power (TP) due to limits in hardware implementation is restricted from 2 dBm to 20 dBm, and for higher levels than 17 dBm, only 1% duty cycle can be used
- Carrier Frequency (CF) is the center frequency that can be programmed in steps of 61 Hz from 137 MHz to 1020 MHz. In some LoRa chips, this frequency range is limited between 860 MHz and 1020 MHz
- Bandwidth (BW): The higher it is, the higher the data rate (thus the shorter time on air); however, sensitivity decreases. LoRa provides three selectable BWs: 125 kHz, 250 kHz, and 500 kHz. Data is sent out at a chip rate equal to the bandwidth in chips per second per Hertz; a bandwidth of 125 kHz corresponds to a chip rate of 125 kcps.
- Coding Rate (CR) refers to the proportion of the transmitted bits that actually carries information. Forward error correction (FEC) techniques are used in LoRa to further increase the receiver sensitivity, which is done by encoding 4-bit data with redundancies into 5-bit, 6-bit, 7-bit, or 8-bit. Using this redundancy will allow the LoRa signal to endure short interferences. The LoRa allowed coding rate values are 4/5, 4/6, 4/7, and 4/8. The more correction bits are used, the easier the data can be corrected, but the duration for the transmission is higher, and the effective data rate is lower.
- Spreading Factor (SF) is the ratio between the symbol rate and chip rate (a LoRa symbol is composed of 2^SF^ chips). Selecting SF from 6 to 12, radio communications with different SF values are orthogonal to each other. SF must be known in advance by the transmitter and receiver in order to detect the message preamble (in other words, to detect at least 4.25 symbols), as the preamble size scales with SF, and there is no single preamble for all SFs.
  A higher SF increases the signal-to-noise ratio (SNR), and thus the sensitivity and range, but also increases the packet airtime. With the same BW, a longer time on air obviously results in less data transmitted per unit of time. Each increase in SF halves the transmission rate, and hence doubles the transmission duration and ultimately energy consumption.

The radio communications under different SF are orthogonal to each other, and thus, the network separation by employing different SF is possible. This way, LoRa symbols can be simultaneously transmitted and received on a same channel without interference. Orthogonality can also be achieved by using different combinations between SF and BW. However, it is necessary to take into account that all the combinations are not orthogonal [93].

Moreover, communication between two elements is only possible if both have the same configuration parameters.

The selection of transmission parameters has an impact on communication performance (transmission range, resilience to interference, and energy consumption of the device). In most situations, it is desirable to balance communication performance and energy consumption, as node devices are battery powered, and it is a goal to maximize lifetime. Obviously, TP has a direct impact on the energy consumption. Another factor determining the energy consumption is the required airtime to transmit a packet, which is dependent on the bitrate and packet size. The bitrate is determined by SF and BW. However, the symbol size of a packet is not only dependent on the payload, but also on SF, BW, and CR.

Investing more energy does not necessarily result in better communication performance. For example, a stronger CR is very energy costly, as packets contain redundant information; however, this only leads to better communication performance in areas with burst interference.

Several configuration options lead to the same energy consumption but impact differently on communication performance. Clearly, it is desirable to select a configuration that minimizes energy consumption while supporting the application requirements in terms of network performance.
